# The structure of quality systems is important to the process and outcome, an empirical study of 386 hospital departments in Sweden

**DOI:** 10.1186/1472-6963-7-104

**Published:** 2007-07-09

**Authors:** Stefan Kunkel, Urban Rosenqvist, Ragnar Westerling

**Affiliations:** 1 Department of Public Health and Caring Sciences, Uppsala University, Uppsala, Sweden

## Abstract

**Background:**

Clinicians, nurses, and managers in hospitals are continuously confronted by new technologies and methods that require changes to working practice. Quality systems can help to manage change while maintaining a high quality of care. A new model of quality systems inspired by the works of Donabedian has three factors: structure (resources and administration), process (culture and professional co-operation), and outcome (competence development and goal achievement). The objectives of this study were to analyse whether structure, process, and outcome can be used to describe quality systems, to analyse whether these components are related, and to discuss implications.

**Methods:**

A questionnaire was developed and sent to a random sample of 600 hospital departments in Sweden. The adjusted response rate was 75%. The data were analysed with confirmatory factor analysis and structural equation modeling in LISREL. This is to our knowledge the first large quantitative study that applies Donabedian's model to quality systems.

**Results:**

The model with relationships between structure, process, and outcome was found to be a reasonable representation of quality systems at hospital departments (p = 0.095, indicating no significant differences between the model and the data set). Structure correlated strongly with process (0.72) and outcome (0.60). Given structure, process also correlated with outcome (0.20).

**Conclusion:**

The model could be used to describe and evaluate single quality systems or to compare different quality systems. It could also be an aid to implement a systematic and evidence-based system for working with quality improvements in hospital departments.

## Background

Clinicians, nurses and managers need tools for building organisations able to cope with emerging medical technologies and methods, while maintaining quality of care [1-3]. Organised systematic quality work, that is, a quality system, can be such a tool[4]. Quality systems can provide data that show politicians, patients, and staff that the departments are working actively and systematically with quality issues. They can also help clinicians and nurses to develop more efficient routines[5,6].

All Swedish hospital departments are required by regulation to have a quality system[7,8]. However, the exact implementation may differ among departments. For instance, in small departments the head of the department may plan and manage this task whereas in large departments the task may be delegated to quality coordinators.

Quality systems have many different names. Quality improvement[3] is a general term that not only often applies to small locally developed systems [9-14] but also to more standardised systems such as Continuous Quality Improvement[15,16]. A more specific term is quality assurance, where quality measures are compared with outcomes in a structured manner, for instance, Total Quality Management or Balanced Scorecard[17,18]. Furthermore, quality accreditation refers to a quality assurance system that adheres to a specific standard, for example ISO9002, and to the fact that the system has been approved by an accreditation agency[19,20].

In this paper, quality systems are broadly defined as organised systematic quality work, covering numerous activities from improvement to accreditation. However, time-limited projects are not included in this definition.

Donabedian's model to analyse quality includes three factors: structure, process, and outcome[21,22]. Structure refers to prerequisites, such as hospital buildings, staff and equipment. Process describes how structure is put into practice, such as specific therapies. Outcome refers to results of processes, for instance, results of therapy.

A qualitative study of department managers and quality co-ordinators, conducted by two of the authors, was the first study, to our knowledge, that applied Donabedian's model to the context of quality systems, rather than to quality itself[4].

In this context, structure refers to available resources, such as time and money for working with quality improvement. It also refers to administration of quality systems, such as documentation of routines and staff support. Process describes quality improvement culture and co-operation within and between professions. Outcome refers to evaluation of goal achievement and development of competence related to quality improvement.

A study of the relationships between structure, process, and outcome could provide information that would benefit clinicians and other professionals, as well as department managers and health policy makers, when developing and working with quality improvement. To reveal such relationships, several aspects and systems would have to be analysed. However, most studies focus on few aspects or few systems[4]. Thus, studies with a broader approach are needed.

The objectives of this study were to analyse whether structure, process, and outcome can be used to describe quality systems, to analyse whether these components are related, and to discuss the implications of these relationships.

## Methods

### Model development

**Figure 1:**
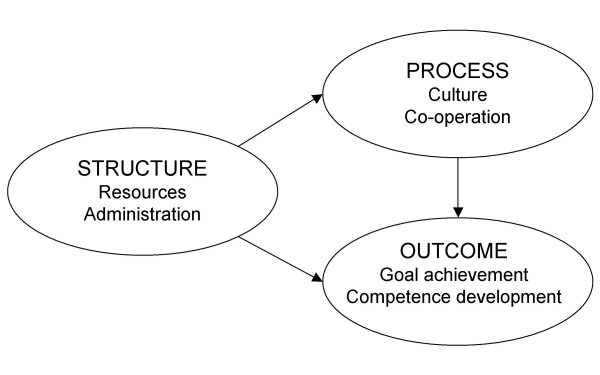
**The proposed model**. Structure is related to process and outcome. Process is related to outcome.

Donabedian has suggested that structure, process, and outcome may be related but that such relationships could be difficult to show[21,22]. A model with relationships between the structure, process, and outcome of quality systems was developed from the results of an interview study (Figure 1)[4]. The model states, for instance, that the more time and money for working with quality improvement (structure), the more positive attitude towards such work (process), and the more regular evaluation of quality related goal accomplishment (outcome).

### The operationalisation of the model

The results from the interview study were used to develop a questionnaire with reflective measures of structure, process, and outcome (Appendix A). The questions have been divided under three headlines in the Appendix only to make it easier for the reader to navigate among them. However, the division into structure, process, and outcome was neither mentioned in the sent out questionnaire nor apparent in the numbering of the questions.

The questionnaire was piloted on informants from the interview study and their responses corresponded well to their interview answers as well as to the developed model. Thus, the pilot questionnaire appeared valid and reliable.

### The data obtained from the questionnaire

A simple random sample of 600 out of 1757 hospital departments in Sweden was provided[23]. The questionnaires were addressed to the head of the respective department, with an option to delegate the task to a quality co-ordinator to increase the response rate if possible. These two groups were chosen since they were thought to possess a more detailed knowledge of quality systems.

Two reminders and a non-responder questionnaire were sent when necessary. The questionnaires were coded, entered, and checked by one of the authors (STK).

Out of the 600 departments, 82 should not have been included in the sample since they were either closed down or associated with larger departments. Thus, responses were expected only from a maximum of 518 departments (Table 1).

**Table 1:** Time of response and reasons not to respond. Frequencies and percentages of sampled departments (n = 600).

	Frequency	Percent
Sampled departments:	600	100
Valid departments	518	86
Closed down departments	52	9
Subordinate departments	30	5
		
Responding clinics:	386	64
Immediate response	261	44
First reminder	79	13
Second reminder	46	8
		
Non-responding departments:	132	22
Reason: Time	33	6
Reason: Relevancy	5	1
Reason: Other, or not stated	25	4
No contact	69	12

In total, 386 valid responses were obtained. The adjusted response rate, 386 out of 518, was 75%.

### The analyses of non-responding departments

Out of the non-responding 132 departments, 63 stated a reason for not responding to the main questionnaire. The most common stated reason was lack of time (Table 1). However, there were no significant differences between responding and non-responding departments in size of hospital (p = 0.07) or speciality of department (p = 0.19).

Partially missing data accounted for only 0.5% of total data and were mostly limited to a single missing value per incomplete case. It has been shown that multiple imputations introduce least estimation bias compared with list-wise deletion[24]. Although it is unlikely that such a small amount of partially missing data would bias the results in any way, the multiple imputations procedure was performed in LISREL as recommended[25].

### The analyses of variables

All variables but two (B2 and B3) had complete response ranges from 1–7 (Appendix B). The frequency distributions were somewhat negatively skewed: responses in the range of 5–7 were more common than in the range of 1–3.

### The assessment of the model

The assessment of a model is a systematic procedure. Each step builds on the previous steps, using progressively more sophisticated statistical methods. This enables the researcher to test the structural model while assuring good validity and reliability[22,23]. The software programme LISREL 8.72 was used for the analyses.

First, an exploratory factor analysis (EFA) was conducted to identify and remove variables that *did not *load significantly onto their intended factor (loading < 0.300, α = 0.05)[22] Three factors were specified for extraction according to the proposed model. Thus, the EFA was exploratory only in the sense that the variables were allowed to load onto all extracted factors.

Second, a confirmatory factor analysis (CFA) was conducted to analyse whether the variables reflected their intended factors and whether the factors could be separated from each other. In CFA, variables are only allowed to load onto the factors specified by the researcher.

Variables that adequately reflect their intended factors should have significant factor loadings (p < 0.05). Reliability should preferably exceed 0.60 and extracted variance should exceed 0.50, to indicate that the variables contribute substantially to the variance of the factors[22,23]. Factors that are adequately separated from each other should have a bivariate factor correlation below 1, indicated by forming 95% confidence intervals[25].

A *good model fit *is indicated by a non significant p-value (p > 0.05), by a low root mean square error of approximation (RMSEA < 0.08), and by a high comparative fit index (CFI close to1.00)[22]. A good model fit means that the model and the data set do not differ significantly.

Third, structural equation modelling (SEM) was used to test the model. In SEM the researcher specifies relationships between some or all of the factors, which is not done in CFA. The results are assessed according to the same measures of model fit used for the CFA[24].

The method of estimation used for the CFA and the SEM was robust maximum likelihood estimation (RMLE) of the covariance matrix and the asymptotic covariance matrix. A weighted least squares estimation (WLSE) of the polychoric correlation matrix and the asymptotic covariance matrix was performed to determine whether the choice of method of estimation might have influenced the results.

Analyses were also conducted to investigate potential differences in the CFA and SEM estimates between groups of responders. Thus, we compared the estimates of the early responder group to those of the whole sample. This type of comparison was also made for the heads of department group. These groups were the only groups that included at least 200 cases as recommeded to be able to reliably perform CFA or SEM.

For some models, such as the proposed model in this paper, CFA and SEM will give identical measures of fit due to the number of factors and the specified relationships. However, in most other cases they will not. Since there is a conceptual difference between CFA and SEM, the SEM was included here to complete the line of argumentation.

## Results

### The exploratory factor analysis (EFA)

Almost all variables (14 out of 18) had factor loadings above 0.300 on their intended factor. However, two process variables (B2 and B3) related to organisational culture did not load significantly at any factor and two outcome variables (C5 and C6) related to the development of competence did not load significantly onto their intended factor (Table 2). They were therefore removed from the following analyses.

**Table 2:** The result of the exploratory factor analysis.

Factors	Factor 1 Structure	Factor 2 Process	Factor 3 Outcome
Variables			
A1	**0.763**	-0.005	-0.038
A2	**0.864**	-0.199	-0.129
A3	**0.578**	0.097	0.006
A4	**0.568**	-0.001	0.149
A5	**0.574**	0.093	0.097
A6	**0.699**	0.039	-0.014
B1	0.153	**0.421**	-0.096
B2	0.286	0.290	-0.029
B3	0.093	0.269	0.035
B4	-0.068	**0.895**	-0.016
B5	0.019	**0.833**	-0.028
B6	-0.050	**0.810**	0.045
C1	**0.344**	0.035	**0.485**
C2	0.108	0.077	**0.768**
C3	-0.018	-0.076	**0.997**
C4	-0.029	0.084	**0.715**
C5	**0.438**	0.176	0.149
C6	**0.422**	0.138	0.060

Significant factor loadings (>0.300) in boldface. The promax rotated pattern matrix is displayed. See Appendix A for questions A1-C6.

### The confirmatory factor analysis (CFA)

The first CFA with 14 variables showed that the structure variable A2 did not reflect structure as expected: the explained variance was only 27%.

The second CFA with 13 variables (A2 excluded), showed that all variables adequately reflected their factors (p < 0.01) and that correlations between factors were significantly less than one (95% confidence interval), thus indicating good construct validity (Table 3). The second CFA was a significantly better model than the first CFA (χ^2 ^difference (11) = 30.3, p = 0.001).

**Table 3:** The result of the confirmatory factor analysis. Model is a reasonable representation of the data (χ ^2 ^(59) = 73.7, p = 0.095, RMSEA = 0.000, and CFI = 1.00). Loadings are unstandardised.

Factor	Variable	Loading	Standard error
Structure	A1	0.88**	0.060
	A3	0.78**	0.065
	A4	0.98**	0.079
	A5	1.19**	0.071
	A6	1.06**	0.070
Process	B1	0.59**	0.069
	B4	1.14**	0.063
	B5	1.21**	0.057
	B6	1.04**	0.065
Outcome	C1	1.12**	0.065
	C2	1.38**	0.066
	C3	1.17**	0.083
	C4	0.96**	0.078

**p < 0.01

95% Confidence intervals for bivariate factor correlations:

Structure – Process: 0.66–0.80

Structure – Outcome: 0.67–0.83

Process – Outcome: 0.54–0.74

Reliability was 0.8 or higher for all factors, which is excellent. Extracted variance was good for process (0.52) and outcome (0.58) but mediocre for structure (0.43).

The model fit measures indicated that the questionnaire reasonably represented the data set (p > 0.05, RMSEA < 0.08, and CFI = 1.00).

### The structural equation modeling (SEM)

**Figure 2:**
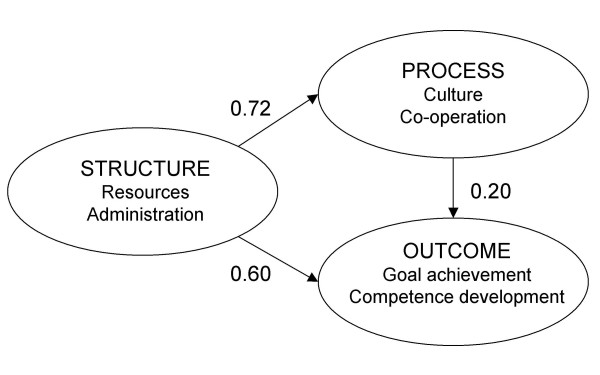
**The structural model**. All relationships are significant (p < 0.05). The model is a reasonable representation of the data (χ^2 ^(59) = 73.7, p = 0.095, RMSEA = 0.000, and CFI = 1.00). The relationship scores can be interpreted as ordinary Pearson correlations.

The structural model was specified according to the proposed model. The hypothesis that the model and the data set did not differ could not be rejected (p = 0.095). Thus, the structural model was accepted (Figure 2).

Structure related strongly with process (0.72) and outcome (0.60). Moreover, process related positively with outcome (0.20). Thus, the total relationship between structure and outcome, including the path via process, was very strong (0.75). Structure and process together explained 58% of the variation in outcome.

The estimates presented above for the whole sample (0.72, 0.60 and 0.20) were very close to those of the early responder group (0.70, 0.58 and 0.23) and those of the heads of department group (0.77, 0.66 and 0.20) (not presented in Figure).

## Discussion

The results of this study strongly indicate that the hypothesised relationships between structure, process, and outcome exist in the context of quality systems.

Structure characteristics, such as available time and staff with quality improvement competence, seem to be strongly related to other aspects of quality systems. Likewise do the existence of a current quality manual with documented routines and task responsibilities, or highly available administrative support, such as secretaries.

Structure seems to be related to process characteristics, such as support from colleagues, in the forms of acceptance towards quality improvement or through active participation in projects.

Structure also seems to be related to outcome characteristics, such as clear and unambiguous goals for the quality system, periodical evaluations of the goals, documentation of the results of the evaluations and feedback of the results to the staff.

Given structure, process also related with outcome. This could indicate that even though structure aspects such as resources and administration are important, work to improve process aspects could further improve outcome. For instance, work to increase support from colleagues could increase the probability that quality efforts get systematically evaluated.

The model suggests that, for instance, if there is enough time to work with quality improvement (structure), there is more support from colleagues (process), and improvements are also evaluated to higher degree (outcome).

### Implications

A systematic and evidence-based approach to quality improvement may increase the chance of effective and efficient use of resources [26-28].

For instance, resources and administration (structure) could be improved by implementing guidelines for quality improvement. Studies show that clinical guidelines, based on evidence rather than opinion, have the potential to promote interventions of proved benefit and discourage ineffective practices [29-32]. Guidelines for quality improvement might have similar effects.

Moreover, culture and co-operation (process) could be enhanced by rewarding good examples and by refraining from punishment when mistakes are reported[33,34]. Co-operative ability could be increased by teamwork training.

Last, evaluation of goal achievement and development of competence (outcome) could benefit from rapid feedback on which measures are effective and which are not[35,36].

The methods used in this paper could also demonstrate how theoretical models can be analysed quantitatively to complement the numerous qualitative studies that exist within the field of health care quality research. For instance, it could be used to analyse the consistency of questions in quality referentials.

However, this study did not show, and could not show due to its design, any links between quality systems and better health outcomes, since no health outcomes were measured.

In further studies, it would be interesting to investigate the implementation of quality systems and relate these processes to departments' history of quality management and overall organisational structure of hospitals. It could also be interesting to evaluate the achievement of specific quality goals because some goals are probably more easily achieved than others.

### Methodological considerations

Although the questionnaire questions may seem general, they were directly developed from the interview study. The CFA showed that most of them clearly reflected their intended factors as indicated by significant factor loadings (sensitivity). The factors could also be clearly separated from each other as shown by factor correlations significantly below 1 (specificity). Each factor was represented by at least three variables, which is considered sufficient to effectively represent most factors[25]. Finally, the good model fit indices also indicated that the questions included in the model were adequate.

Some of the questions were about potentially sensitive matters, such as B3 which was about attitudes to incident reporting. However, these questions were removed from the analyses since the CFA indicated that they were not adequate reflections of their factors. Thus, the potential subjectivity or sensitivity of these questions could not have affected the results of the SEM.

A minimum sample size of 200 is recommended for doing CFA or SEM if the proposed model is not overly complex and if a maximum likelihood type of estimation is used[24] Thus, the sample size of 386 was deemed adequate since it was 1.9 times the minimum recommendation and since the analysed models were not complex. Moreover, the particular type of estimation chosen for the analyses (RMLE) is distribution independent and thus does not require data multivariate normality[25].

Only the early responder and heads-of-department groups had the recommended minimum of 200 cases. Thus, it was decided not to analyse differences between groups, for instance, between departments in hospitals of different sizes. However, large differences between groups of cases would have lead to the rejection of the tested model[25].

Although the response rate was high (75%), bias created by non-responders cannot be ignored as a possibility. The quality systems of non-responding clinics may have differed from the quality systems of responding clinics in such ways as to affect the probability of responding to the questionnaire. Moreover, non-responders might have had different attitudes towards the questionnaire or towards quality systems in general than responders had.

The data missing from non-responding departments could also potentially affect the robustness of the SEM estimates and the model. However, the SEM estimates and the model fit indices passed the tests with good margins according to recommended statistical standards[24,25]. Furthermore, the SEM estimates and model fit indices of the early responder group did not differ from those of the whole sample. Moreover, there were no significant differences in composition between responders and non-responders with regard to hospital size or department speciality. Thus, the results appear stable and independent of the time of response.

The estimated parameters obtained by the RMLE and the WLSE did not differ significantly and did not change the conclusions of this study. Thus, the results appear stable and independent of the choice of method of estimation.

It is possible that heads of departments would answer the questions differently compared with employees without managerial positions. However, how heads of departments and quality coordinators responded did not differ significantly.

An advantage of using CFA and SEM is that these methods analyse both the significance of the relationships and the model fit. Thus, it can be justified to include even small significant relationships, such as the one between process and outcome, as long as model fit improves and they have reasonable interpretations.

In theory, the structure of quality systems affects process and outcome. Since this is a cross-sectional study it is important to be careful when discussing causal relationships. However, structure is strongly related to the other two aspects, which may suggest that it is more important.

This is to our knowledge the first large quantitative study that applies Donabedian's model to quality systems.

### Ethical considerations

Formal ethical approval of this study was not needed. However, steps were naturally taken to assure compliance with general ethical principles for conducting research. Respondents were informed of the purpose of the study and that their responses would be kept confidential. Responding to the questionnaire was voluntary and no gifts or other rewards were promised responders. The research project plan was approved by the Faculty of Medicine at Uppsala University (MedFarm 2003/1313-C4:2).

## Conclusion

The structural model with relationships between structure, process, and outcome reasonably represented quality systems at hospital departments. Relationships between structure and process (0.72), structure and outcome (0.60), and process and outcome (0.20) were found as expected according to theory. This would indicate that, for instance, adequate resources and administration may play an important role in systematic quality work.

## Appendices

See Table 4 for Appendix A. The questionnaire. 

**Table 4:** Appendix A. The questionnaire

Labels	Questions
Structure	
A1	Do the clinic's employees and managers have time to work with quality improvement?
A2	Are there enough employees to implement new quality improvement methods?
A3	Do the clinic's employees and managers have the right competence for working with quality improvement?
A4	Are the clinic's routines documented in a quality manual or similar? (such as filing system with routines for treatment, quality development or evaluation.)
A5	Are there documents on which employee should do what in quality improvement?
A6	Does the clinic have administrative support for working with quality? (such as access to computers, secretaries or advice on how to work with quality improvement.)
Process	
B1	In general, is it easy to get support from the clinic's colleagues when trying to implement new organisational improvements?
B2	In general, is it easy to get support from the clinic's managers when trying to implement new organisational improvements?
B3	Are the clinic's employees positive to reporting incidents?
B4	Are members of all professions participating actively in working with quality?
B5	Are most of the clinic's employees participating actively in working with quality?
B6	Do members of different professions co-operate regarding quality related work?
Outcome	
C1	Does the clinic have precise quality related goals for the clinic?
C2	Does the clinic periodically evaluate if the quality related goals are accomplished?
C3	Are the results of the evaluations documented?
C4	Are the results of the evaluations communicated to the employees?
C5	Are new employees introduced to the clinic's routines for working with quality?
C6	Do the clinic's employees get opportunities to educate themselves in how to work with quality improvement?

See Table 5 for Appendix B. The frequency distribution for each of the measured variables.

**Table 5:** Appendix B. The frequency distribution for each of the measured variables.

Alternative	1	2	3	4	5	6	7
	%	%	%	%	%	%	%
Structure							
A1	1.0	11.9	18.9	24.4	29.3	12.2	2.3
A2	3.4	14.5	21.8	23.1	19.9	15.0	2.3
A3	0.3	3.9	14.5	21.8	26.9	27.5	5.2
A4	1.6	7.8	8.8	16.6	20.5	24.9	19.9
A5	2.3	11.7	13.2	17.1	20.5	19.7	15.5
A6	1.8	8.3	9.8	14.0	22.0	28.5	15.5
							
Process							
B1	0.5	6.2	13.5	18.1	29.8	25.1	6.7
B2	0.0	1.6	3.9	11.7	25.4	42.5	15.0
B3	0.0	4.4	7.8	14.5	25.6	35.2	12.4
B4	0.5	6.5	6.7	17.1	25.1	28.5	15.5
B5	1.6	9.8	15.3	22.3	25.1	17.9	8.0
B6	0.3	4.7	7.3	18.7	25.4	29.3	14.5
							
Outcome							
C1	1.6	2.8	10.6	17.4	24.1	31.1	12.4
C2	3.1	8.3	14.0	14.5	25.9	20.7	13.5
C3	2.3	8.0	12.7	12.2	25.4	22.8	16.6
C4	1.8	4.9	8.8	14.5	24.1	29.3	16.6
C5	2.1	7.3	12.4	24.4	22.8	22.0	9.1
C6	0.8	6.7	11.4	19.7	30.1	25.9	5.4

Response alternatives correspond to: 1 = "to a low degree", 7 = "to a high degree"

## Pre-publication history

The pre-publication history for this paper can be accessed here:


